# Quality of IVF status registration in the Estonian Medical Birth Registry: a national record linkage study

**DOI:** 10.1186/s12978-018-0575-7

**Published:** 2018-08-08

**Authors:** Kärt Allvee, Mati Rahu, Kai Haldre, Helle Karro, Kaja Rahu

**Affiliations:** 1 Estonian Medical Birth Registry, National Institute for Health Development, Hiiu 42, 11619 Tallinn, Estonia; 2 Department of Epidemiology and Biostatistics, National Institute for Health Development, Hiiu 42, 11619 Tallinn, Estonia; 3 Centre for Reproductive Medicine, West Tallinn Central Hospital Women’s Clinic, Sõle 23, 10614 Tallinn, Estonia; 4 Department of Obstetrics and Gynecology, Institute of Clinical Medicine, University of Tartu, L. Puusepa 8, 51014 Tartu, Estonia; 5 Tartu University Hospital’s Women’s Clinic, L. Puusepa 8, 51014 Tartu, Estonia

**Keywords:** Birth registry, Data quality, Embryo transfer, In vitro fertilization, Record linkage

## Abstract

**Background:**

Information regarding in vitro fertilization (IVF) as a pregnancy risk factor (yes/ no) is stored in each birth record of the Estonian Medical Birth Registry (EMBR). This study aimed to assess the validity of registration of newborns’ IVF status in the EMBR.

**Methods:**

To identify the newborns conceived by IVF, the birth records in the EMBR were compared to individual records on the embryo transfer procedures in the Estonian Health Insurance Fund (EHIF) database as a reference. Maternal age was restricted to 40 years, the age limit for IVF treatment covered by the EHIF. The embryo transfer procedures, that dated up to eight weeks before pregnancy, were additionally checked in the infertility treatment clinics. The validity of IVF status was measured by sensitivity, specificity, positive and negative predictive values (PPV and NPV). Relative risk (RR) of unrecorded IVF status among IVF mothers by socio-demographic characteristics and birth plurality was estimated using modified Poisson regression models.

**Results:**

There were 3198 newborns identified as conceived by IVF in the EMBR in 2005–2014. Eight of them were incorrectly entered as born after IVF. The record linkage with the EHIF database revealed 1014 newborns with unrecorded IVF status in the EMBR. A total of 4204 newborns were verified as conceived by IVF, 24.1% of them were not categorized as born after IVF. The sensitivity of the IVF status registration was poor (75.9%), specificity (100.0%), PPV (99.8%) and NPV (99.3%) were high. The misclassifications were significantly more common among mothers of younger age or non-Estonians or with singleton birth.

**Conclusion:**

Information based on mother’s self-report or her antenatal chart does not accurately identify the newborn’s IVF status. The lack of a specialized country-wide assisted reproductive technology register in Estonia requires routine record linkage of the EMBR, EHIF and the infertility treatment clinics’ databases to obtain adequate information regarding IVF status in the EMBR. Electronic record linkages between databases would help considerably to improve the validity of data to be used in medical decision making, in research and for statistical purposes.

## Plain English summary

Birth records in the Estonian Medical Birth Registry (EMBR) include information on the IVF status of newborns relying on mothers’ self-report or their antenatal charts. As there is no mandate to sanction data exchange between the EMBR and infertility treatment clinics, current study aimed to assess the validity of the IVF status registration in the registry via linkage records of the EMBR and Estonian Health Insurance Fund (EHIF) reimbursement database as a reference. Solidarity based EHIF covers unlimited number of IVF cycles for all women aged 40 years or less. The embryo transfers resulting in birth were identified using the mother’s personal identification number as the key. If necessary, additional verification in the infertility treatment clinics was performed. According to the EMBR data, 146,526 infants with maternal age up to 40 years were born to Estonian residents in 2005–2014. A total of 4204 newborns were verified as conceived by IVF; 1014 newborns had lacked the IVF status on the birth record. The sensitivity of the IVF status registration was poor (75.9%). The IVF status was better recorded for mothers of the older age group (35–40 years) or with a multiple birth, because they were more likely to have IVF pregnancy and were more carefully questioned. As far as Estonia lacks a specialized country-wide assisted reproductive technology register, record linkages of the EMBR, EHIF and the infertility treatment clinics’ databases are requested to obtain adequate information on the IVF status of the newborns in the EMBR.

## Background

The 2014 Estonian Women’s Health Questionnaire Survey indicated that 15.8% of the sexually active respondents at 18–44 years of age had experienced infertility, and 13.4% of them reported the use of in vitro fertilization (IVF) to have a child [1]. The proportion of births after IVF in Estonia is similar to the European average, which ranges from 2% to 4% [2]. It is important to obtain reliable data on the IVF status of a newborn to monitor IVF pregnancy outcomes and long-term health of children conceived by IVF. As there is currently no specialized country-wide assisted reproductive technology register in Estonia, sole information regarding IVF as a pregnancy risk factor (yes/ no) (defined as embryo transfer after conventional in vitro insemination or intracytoplasmic sperm injection [3]) is stored in each birth record of the Estonian Medical Birth Registry (EMBR).

The unique personal identification numbers (PINs) of the newborn and his/ her mother are registered in the EMBR and allow for record linkage [4]. The IVF status is recorded on the birth notification form by the mothers’ self-report or using her antenatal chart. As a result, omissions are common because there is no routine information exchange between the EMBR and the infertility treatment clinics. However, IVF treatment is generously covered by the Estonian Health Insurance Fund (EHIF). Therefore, majority of the IVF procedures are recorded in the reimbursement database with women’s PINs [5].

The previous study that estimated effectiveness and costs of IVF treatment in Estonia using linkage of the EMBR and EHIF records, reported 15.5% fewer IVF entries in the EMBR in 2006–2011 than were based on the EHIF data. A birth in the EMBR was considered an IVF birth if the embryo transfer procedure was performed within 42 weeks before delivery [6].

Current study aimed to assess the validity of registration of newborns’ IVF status in the EMBR by linking the EMBR and EHIF records.

## Methods

We assessed the validity of registration of newborns’ IVF status in the EMBR by comparing information about it on the records of the EMBR and of the EHIF.

The EMBR, currently under the responsibility of the National Institute of Health Development, was established in 1991 and collects data on all infants born in Estonia [4, 7]. The hospitals with a maternity unit are obliged to notify births to the EMBR. A paper birth notification form is filled in for every newborn (live or stillbirth). This form includes the name, PIN, date of birth, ethnicity, education and place of residence of the mother and the father; the mother’s marital status, reproductive history, pregnancy risk factors, complications during pregnancy and delivery, labor interventions, mode of delivery and birth plurality; the newborn’s PIN, date of birth, gender, gestational age, birthweight, length, condition at birth, health indicators and status by the end of the first week. In the EMBR, data from birth notifications are key-entered and electronic records are created [8].

The EHIF, founded in 2001, is the only institution in Estonia that administers the national compulsory solidarity-based health insurance system [5]. The fund covers the costs of health services provided to insured persons, who constitute 95% of the Estonian population. The EHIF maintains an electronic health insurance database that consists of reimbursement claims for medical care. The reimbursement claims are sent to the EHIF electronically, the records in the database are stored at the individual level. Each record contains the name and PIN of the patient, medical diagnosis codes, provided service codes, service provider, amount requested for reimbursement and other associated information [9]. The age limit for IVF treatment covered by the EHIF was raised from 35 to 40 years in 2005, and unlimited number of IVF cycles is subsidized since 2008 [10].

**Fig. 1:**
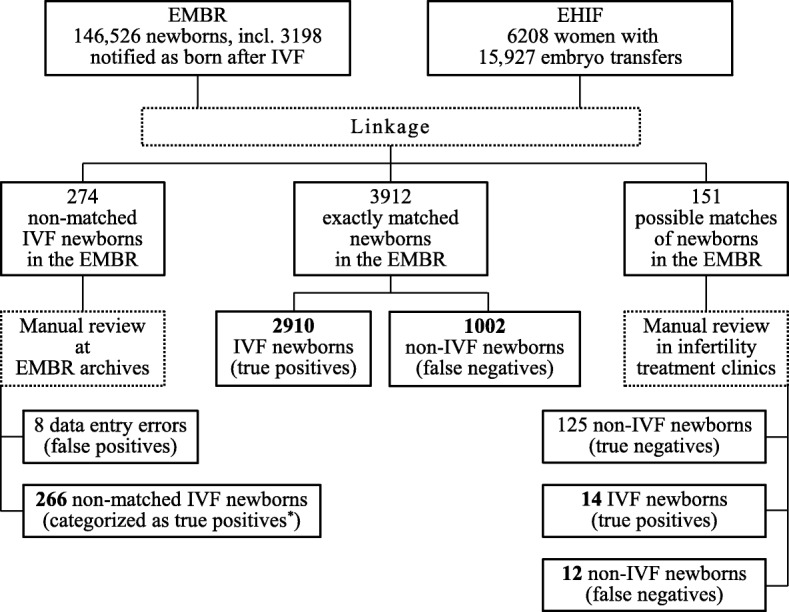
Data collection process for estimation validity of IVF status registration in the Estonian Medical Birth Registry (EMBR), mothers aged ≤40 years, 2005–2014. Sum of the numbers formatted as bold gives the number of newborns conceived by IVF in the EMBR. ^*^ Embryo transfers were not covered by the Estonian Health Insurance Fund (EHIF)

The validity of registration of newborns’ IVF status in the EMBR was measured by comparing the birth records in the EMBR and the corresponding embryo transfer records in the EHIF. Thus, the EHIF was treated as a reference. Individual records of the embryo transfers in 2004–2014 (earlier data were not available) including woman’s PIN, date of the procedure and name of the clinic were obtained from the EHIF database (6208 women with 15,927 embryo transfers in all five clinics providing the service) (Fig. 1). The embryo transfers resulting in birth were identified via linkage of these records to the EMBR data using the mother’s PIN as the key. As the EHIF data were available since 2004 and considering the age limit for IVF treatment, we selected the birth records in the period of 2005–2014 and maternal age was restricted to 40 years (146,526 infants born to Estonian residents).

We compared the date of embryo transfer with the date of the beginning of pregnancy (calculated by subtracting gestational age from the date of birth in the EMBR). The beginning of pregnancy after IVF (comparable to that of spontaneously conceived newborns) dates 2–3 weeks prior to the date of the embryo transfer [11, 12]. Thus, all births to mothers with date of the embryo transfer procedure within the pregnancy period were categorized as births after IVF.

Whereas the reimbursement claims of the EHIF may contain inaccuracies in documenting the date of the embryo transfer procedure, additional validation of the IVF status was undertaken in the infertility treatment clinics. In site visits, we wished to ascertain that embryo transfers performed during eight weeks before the beginning of pregnancy (by EHIF data), were not related to the pregnancies under study, and these newborns were spontaneously conceived after unsuccessful IVF treatment. Repeated embryo transfers, except the last one before the pregnancy that resulted in birth, were not taken into consideration. Thus, first, a list of eligible mothers (PIN, date of embryo transfer, pregnancy period, child’s date of birth, the name of the clinic) was printed out. Second, in the infertility treatment clinics, the list of these mothers was visually compared with a screen output of their clinical data. As a result, IVF and non-IVF newborns were distinguished (Fig. 1). All births notified as after IVF but without corresponding embryo transfer record in the EHIF database were compared with paper birth notifications at the archives of the EMBR.

The validity of the IVF status in the EMBR was evaluated by determining the sensitivity, specificity, positive predictive value and negative predictive value, all often used in validation of health databases [13–16]. The sensitivity shows how accurately the IVF status has been recorded in the EMBR (true-positive cases divided by true-positive plus false-negative cases). The specificity demonstrates the ability of the EMBR to record non-IVF births (true negative cases divided by true-negative plus false-positive cases). The positive predictive value indicates how likely it is that the birth after IVF in the EMBR is registered correctly (true positive cases divided by true-positive plus false-positive cases). The negative predictive value reveals how likely is the non-IVF birth correct in the EMBR (true negative cases divided by true-negative plus false-negative cases).

Additionally, we calculated the relative risks (prevalence proportion ratios; RR) with 95% confidence intervals (CI) of unrecorded IVF status among IVF mothers by available socio-demographic characteristics and birth plurality. Modified Poisson regression models (with a robust error variance) [17] included maternal age (< 35/ 35–40 years), education (higher/ secondary or less), ethnicity (Estonian/ non-Estonian), marital status (married/ other), and plurality (singleton/ multiple birth). Age group of 35–40 years, higher education, Estonian ethnicity, being married and multiple birth were selected as reference categories. In a separate model we tested whether the data quality on IVF status was higher if the infertility treatment and delivery occurred in the same clinic; on this occasion, 167 records with unknown data on the infertility treatment clinic were excluded.

Visual FoxPro 9.0 (Microsoft Corporation, Redmond, WA, USA) was used for database management and linkages, and Stata 14 (StataCorp LP, College Station, TX, USA) for validity calculations and statistical modeling.

## Results

According to the EMBR data for 2005–2014, 3198 infants were born to mothers aged 40 years or less and identified as conceived by IVF. The record linkage with the EHIF database resulted in 3912 newborns in the EMBR with the corresponding embryo transfer record (the date of the embryo transfer procedure was within the pregnancy period), whereas 1002 of them were with unrecorded IVF status, i.e., erroneously categorized as conceived spontaneously (false negatives) (Fig. 1). Among them were 778 singletons, 230 twins and 6 triplets. From the 151 additional comparisons of birth records with the embryo transfer procedure before the beginning of pregnancy in the infertility treatment clinics, 14 newborns were verified as born after IVF (true positives) and 12 newborns notified with non-IVF status were found to be conceived by IVF (false negatives) (Fig. 1).

The record linkage revealed 274 newborns notified to be born after IVF in the EMBR with no corresponding embryo transfer record in the EHIF database. These records were examined at the EMBR paper archives and it was found that during data entry in the EMBR eight newborns have been incorrectly categorized as born after IVF (data entry errors; false positives) (Fig. 1). The rest of 266 newborns were categorized as born after IVF (true positives) as there could be a few IVF mothers who covered their treatment themselves (see Discussion). A total of 4204 infants born to 3435 mothers were verified as conceived by IVF; 1014 infants (24.1%) born to 895 mothers (26.1%) had lacked the IVF status on the birth record.

The 1014 false negative IVF entries resulted in poor sensitivity of the registration of IVF status (75.9%; 95% CI 74.6–77.2); specificity (100.0%), PPV (99.8%) and NPV (99.3%) were high (Table 1).

**Table 1:** Number of IVF and non-IVF newborns in the Estonian Medical Birth Registry (EMBR) compared to the Estonian Health Insurance Fund (EHIF) data, and the IVF registration validity measures with 95% confidence intervals (CI), 2005–2014

EMBR	EHIF database (a reference)		
	IVF newborn	Non-IVF newborn	Total
IVF newborn	3190	8	3198
Non-IVF newborn	1014	142,314	143,328
Total	4204	142,322	146,526
Sensitivity (%) = 75.9 (95% CI 74.6–77.2)			
Specificity (%) = 100.0 (95% CI 100.0–100.0)			
Positive predictive value (%) = 99.8 (95% CI 99.5–99.9)			
Negative predictive value (%) = 99.3 (95% CI 99.2–99.3)			

An unrecorded IVF status was significantly more common among mothers of younger age (< 35 years) or with non-Estonian ethnicity or with a singleton birth (Table 2). The misclassification did not depend on mother’s education or marital status. We did not observe higher registration quality of IVF status if the infertility treatment and delivery occurred in the same clinic (“not the same” as reference: adjusted RR = 0.96; 95% CI 0.86–1.07).

**Table 2:** Relative risk (RR) with 95% confidence interval (CI) of unrecorded IVF status in the Estonian Medical Birth Registry by maternal characteristics, 2005–2014

Maternal characteristic	No. of IVF mothers		RR (95% CI)	
	Unrecorded IVF status	Total	Crude	Adjusted^*^
Age (years)				
< 35	587	2146	1.14 (1.02–1.29)	1.17 (1.04–1.32)
35–40	308	1289	1	1
Education				
Higher	483	1846	1	1
Secondary or less	412	1589	0.99 (0.89–1.11)	0.96 (0.86–1.08)
Ethnicity				
Estonian	627	2534	1	1
Non-Estonian	268	901	1.20 (1.06–1.36)	1.20 (1.06–1.36)
Marital status				
Married	500	1925	1	1
Other	395	1510	1.01 (0.90–1.13)	1.05 (0.93–1.18)
Plurality				
Singleton birth	778	2693	1.83 (1.54–2.19)	1.85 (1.55–2.21)
Multiple birth	117	742	1	1

^*^Adjusted for all other characteristics in the table

## Discussion

This study presents the results of the record linkages of the EMBR and the EHIF data to evaluate births following embryo transfer procedures. Although a decade ago the general data quality of the EMBR has been found to be acceptable [18], the currently estimated sensitivity of 75.9% of IVF status registration is worrying despite of high values of other validity measures.

Similarly, nearly a quarter of missing IVF entries was found in the Finnish Medical Birth Registry after linkage with insurance reimbursement files, drug prescriptions and IVF statistics [19]. Given that data from medical birth registries are often used to compare perinatal outcome and subsequent health of IVF babies with that of their counterparts conceived spontaneously [20–22], under-registration of IVF status biases effect measures.

The IVF status recorded on the antenatal chart is completed by the doctor or midwife at the first antenatal visit. The source of this information can be the electronic health record or self-report of the pregnant woman. In the cases where the IVF status is not mentioned on the antenatal chart, it is based on the mother’s self-report after the delivery as “yes/ no” answer to the question: “Did you have IVF pregnancy?” However, the question is not always asked and the mother can refuse to reveal her IVF status. Additionally, if the abbreviation IVF is used, it may lead to misunderstanding. Expectedly, the IVF status was better registered among mothers aged 35–40 years, and mothers with multiple birth, because they were more likely to have IVF pregnancy and were more carefully questioned. The higher under-recording of IVF status among non-Estonians could be due to language or poor attention to the question in the clinics located in the regions with a high proportion of non-Estonians (northeastern Estonia). We found equally low-quality registration of IVF status when the birth occurred in the same clinic that performed IVF treatment, or when the birth took place in another clinic. This finding indicates that there is limited information exchange even between the departments of the same clinic. Thus, the validity of the IVF entries in the EMBR has so far relied on self-report of mothers and on the accuracy of the medical personnel when completing the antenatal chart.

However, there were 266 records of the newborns after IVF in the EMBR with no corresponding embryo transfer record in the EHIF database verifying the IVF status. We had no reason to classify them as non-IVF newborns (false positives) as their mothers could have paid for the treatment themselves because they did not have health insurance; or because of a will to have out-of-pocket service; or because they have used IVF services in other countries; or because only the limited number of IVF cycles were covered by the EHIF prior to 2008 [10]. On the other hand, we cannot deny that among newborns notified in the EMBR as spontaneously conceived could be a few newborns after IVF (false negatives). On this occasion, mothers who have had out-of-pocket service have not disclosed their IVF status. Even so, very small number of these cases would not change the results notably.

The previous linkage study [6] with somewhat different methodology (incorporating maternal age above 40 in the EMBR, not considering individual length of gestation, and without verification of an uncertain IVF status in the infertility treatment clinics) and covering shorter period, found 15.5% fewer newborns after IVF in the EMBR than were expected from the EHIF data. This estimate was much smaller than the percentage of unrecorded IVF entries in the EMBR in the current study. It should be noticed, that in the previous study, only the difference in the number of newborns after IVF based on the EHIF data and the number of newborns after IVF in the EMBR were compared not considering individual unrecorded IVF entries in the EMBR. These outcomes of the two studies would have been of the same magnitude if all newborns after IVF in the EMBR were reflected in the EHIF database. The discrepancy is explained by the number of newborns after IVF in the EMBR with no confirming record in the EHIF database.

## Conclusions

It is difficult to obtain complete data for newborns’ IVF status in the EMBR by exclusively relying on mother’s self-reported information or her antenatal chart. As far as we do not have a specialized country-wide assisted reproductive technology database in Estonia, the routine linkages between the EMBR, the EHIF database, and data from infertility treatment clinics would be helpful. Furthermore, collecting valid data for IVF pregnancies is important due to the common use of assisted reproductive technology [23]. The increasing number of digital health databases gives better than ever possibilities for additional data quality control. Electronic record linkages between databases would help considerably to improve the validity of data to be used in medical decision making, in research and for statistical purposes.

## Abbreviations

CI: confidence interval
EHIF: Estonian Health Insurance Fund
EMBR: Estonian Medical Birth Registry
IVF: in vitro fertilization
PIN: personal identification number
RR: relative risk
